# Ribozyme-mediated inactivation of mutant K-ras oncogene in a colon cancer cell line

**DOI:** 10.1054/bjoc.2000.1363

**Published:** 2000-08-17

**Authors:** T Tokunaga, T Tsuchida, H Kijima, K Okamoto, Y Oshika, N Sawa, Y Ohnishi, H Yamazaki, S Miura, Y Ueyama, M Nakamura

**Affiliations:** Department of Pathology, Tokai University School of Medicine, Bohseidai, Isehara, Kanagawa, 259–1193; Second Department of Internal Medicine, National Defence Medical Collage, Namiki 3–2, Tokorozawa, Saitama, 359; Central Institute for Experimental Animals, Nogawa 1430, Kawasaki, Kanagawa, 213, Japan

## Abstract

Mutation of c-K-ras oncogene is an important step in progression of colon cancer. We used a hammerhead ribozyme (KrasRz) against mutated K-ras gene transcripts (codon 12, GTT) to inactivate mutant K-ras function in the colon cancer cell line SW480, harbouring a mutant K-ras gene. The β-actin promoter-driven KrasRz sequence (pHβ/KrasRz) was introduced into these cells (SW480/KrasRz), and we evaluated its effects on growth of the colon cancer. The gene expression of angiogenesis-related molecules (vascular endothelial growth factor and thrombospondin) was also estimated in SW480/KrasRz. KrasRz specifically and efficiently cleaved the mutant K-ras mRNA but not wild-type mRNA in vitro. SW480/KrasRz showed decreased growth rate under tissue culture conditions (*P*< 0.01, Dunnett’s test). The xenotransplantability of SW480/KrasRz (XeSW480/KrasRz) was significantly decreased in nude mice (*P*< 0.05, Fisher’s exact test). Tumour volume of the xenografts XeSW480/KrasRz was significantly smaller than that of XeSW480/DisKrasRz (*P*< 0.01, Dunnett’s test). Gene expression of VEGF was suppressed in SW480/KrasRz, while TSP1 gene expression was enhanced. The SW480/KrasRz cells showed apoptosis-related features including nuclear condensation and DNA fragmentation. These results suggested that the hammerhead ribozyme-mediated inactivation of the mutated K-ras mRNA induced growth suppression, apoptosis and alteration of angiogenic factor expression. © 2000 Cancer Research Campaign


British Journal of Cancer (2000) 83(6), 833–839
© 2000 Cancer Research Campaign
doi: 10.1054/ bjoc.2000.1363, available online at http://www.idealibrary.com on IDEAL®

### Ribozyme-mediated inactivation of mutant K-ras oncogene in a colon cancer cell line

**T Tokunaga[1,2], T Tsuchida[1], H Kijima[1], K Okamoto[3], Y Oshika[1], N Sawa[3], Y Ohnishi[3], H Yamazaki[1], S Miura[2], Y Ueyama[1] and M Nakamura[1]**

[1]Department of Pathology, Tokai University School of Medicine, Bohseidai, Isehara, Kanagawa 259–1193; [2]Second Department of Internal Medicine, National Defence Medical Collage, Namiki 3–2, Tokorozawa, Saitama 359; [3]Central Institute for Experimental Animals, Nogawa 1430, Kawasaki, Kanagawa 213, Japan

**Summary** Mutation of c-K-ras oncogene is an important step in progression of colon cancer. We used a hammerhead ribozyme (KrasRz) against mutated K-ras gene transcripts (codon 12, GTT) to inactivate mutant K-ras function in the colon cancer cell line SW480, harbouring a mutant K-ras gene. The β-actin promoter-driven KrasRz sequence (pHβ/KrasRz) was introduced into these cells (SW480/KrasRz), and we evaluated its effects on growth of the colon cancer. The gene expression of angiogenesis-related molecules (vascular endothelial growth factor and thrombospondin) was also estimated in SW480/KrasRz. KrasRz specifically and efficiently cleaved the mutant K-ras mRNA but not wild-type mRNA in vitro. SW480/KrasRz showed decreased growth rate under tissue culture conditions ($P < 0.01$, Dunnett's test). The xenotransplantability of SW480/KrasRz (XeSW480/KrasRz) was significantly decreased in nude mice ($P < 0.05$, Fisher's exact test). Tumour volume of the xenografts XeSW480/KrasRz was significantly smaller than that of XeSW480/DisKrasRz ($P < 0.01$, Dunnett's test). Gene expression of VEGF was suppressed in SW480/KrasRz, while TSP1 gene expression was enhanced. The SW480/KrasRz cells showed apoptosis-related features including nuclear condensation and DNA fragmentation. These results suggested that the hammerhead ribozyme-mediated inactivation of the mutated K-ras mRNA induced growth suppression, apoptosis and alteration of angiogenic factor expression.
© 2000 Cancer Research Campaign

**Keywords**: colon cancer; K-ras oncogene; angiogenic factor; ribozyme

Human colon cancer can be well screened using a multidisciplinary panel (Jednak and Nostrant, 1998), although it is the third most commonly diagnosed cancer and the second leading cause of cancer death in Japanese women. Initiation and progression of colon cancer are related to a series of mutations involving several oncogenes (Cho and Vogelstein, 1992). The growth of solid tumours beyond a certain critical volume and the development of tumour metastasis is related to the establishment of vasculature that nourishes the tumour cells. Recently, various studies have shown that some tumour-associated genes are involved in the regulation of angiogenesis. For example, mutant p53 potentiates protein kinase C induction of vascular endothelial growth factor A (VEGF) (Kieser et al, 1994). Other human tumour suppressor genes including the Von Hippel Lindaw (VHL) gene and chromosome 10q also influence the angiogenesis-related factors (Hsu et al, 1996; Siemeister et al, 1996). Alterations of the K-ras oncogenes are early events in the adenoma–carcinoma sequence (Saraga et al, 1997). A single amino-acid substitution in codon 12, 13 or 61 of the ras oncoprotein activates its p21 gene products, and may be related to tumour aggressiveness (Andreyev et al, 1998). The majority of colon cancers harbour point mutations in codon 12 of the K-ras oncogene. The mutated ras oncoproteins are thought to alter the cellular signal transduction pathways (Matsuguchi and Kraft, 1998) and to affect neoplastic growth of colon cancer. Mutations that result in constitutive activation of the K-ras gene are associated with reduction of colon cancer cell proliferation and angiogenesis (Rak et al, 1995). K-ras oncogene-dependent VEGF expression is necessary for progressive in vivo growth of cancer cell lines (Okada et al, 1998). There is evidence that the ras oncogene may be correlated with apoptosis (Ward et al, 1997). Oncogenic ras triggers cell suicide through activation of a caspase-independent cell death programme in human cancer cells (Chi et al, 1999). Dominant negative mutants of ras oncogenes have been shown to enhance apoptosis in erythroleukaemia cells. Therefore, the mutated K-ras gene can become a specific modulator of suppression of aggressiveness in colon cancer cells.

*Received 29 November 1999*
*Revised 27 April 2000*
*Accepted 1 May 2000*

*Correspondence to*: M Nakamura

Specific gene modifications have been made using oligonucleotides, and this has been shown to be an important strategy for suppressing activated oncogenes. This strategy using oligonucleotides including triplex DNAs, antisense nucleotides and ribozymes (catalytic RNAs) offers the potential to achieve oncogene ablation at a proximal level of gene expression (Kijima et al, 1998). Ribozymes were initially discovered in the group 1 intervening sequence in the pre-RNA of *Tetrahymena thermophilia* (Cech et al, 1981). Highly specific activities of hammerhead ribozymes were demonstrated and shown to inhibit the expression of specific genes by targeting their mRNAs. The ribozymes are considered to be better inhibitors of gene expression than non-catalytic oligonucleotides. Ribozyme strategies have advantages because of their site-specific cleavage activities and catalytic potential (Kijima et al, 1995). Recently, ribozymes were reported to be effective against various oncogenes (e.g. c-fos, ras, bcr-abl) (Scanlon et al, 1991; Bouffard et al, 1996) and the drug resistance genes (e.g. MDR1). We also demonstrated that anti-mutated gene ribozymes effectively suppressed the expression of targeted genes and caused reversal of the malignant phenotype in cancer cells (Ohta et al, 1996; Tsuchida et al, 1998; Yamazaki et al, 1998).

In this study, we used a hammerhead ribozyme designed to specifically cleave mutated K-ras oncogene mRNA in human colon cancer cell lines carrying K-ras point mutations. We examined the effectiveness of this ribozyme for mutation-specific inhibition of K-ras oncogene resulting in inhibition of angiogenic factor and growth suppression in a colon cancer cell line. Here, we report the therapeutic potential of anti-K-ras ribozyme against human colon cancer.

### MATERIALS AND METHODS

### Cells

The SW480 human colon cancer cell line harbouring a mutant c-K-ras oncogene was obtained from American Tissue Culture Collection (#ATCC CCL-228; Rockville, MD, USA). The cell line was maintained in Leibobitz's L-15 medium (Sigma, St. Louis, MO, USA) containing 10% fetal bovine serum (Sigma), as well as 100 IU $ml^{-1}$ penicillin and 100 μg $ml^{-1}$ streptomycin (Gibco BRL, Gaithersburg, MD, USA). Total RNAs were extracted from SW480 cells and the transformed cell line by a single-step procedure using guanidinium isothiocyanate and acid phenol/chloroform.

### Preparation of anti-K-ras ribozymes

A hammerhead type ribozyme (KrasRz) was synthesized by 30 cycles of PCR (Perkin-Elmer, Cetus, USA) with the following primers: 5′-TAATACGACTCACTATAGCTACGCC-CTGATGAGTCCGTGA-3′ (RzS) and 5′-GAGCTGTTTCGTC-CTCACGGACT-3′ (RzA) (Figure 1A). A disabled hammerhead ribozyme (dis-KrasRz) was also synthesized by PCR with the following primers: 5′-TAATACGACTCACTATAGCTACGCCC-TAATGAGTCCGTGA-3′ (DRzS) and 5′-GAGCTGTTTCGTC-CTCACGGACT-3′ (RzA). The disabled ribozyme contained a single base exchange (G to A, underlined, see above; Figure 1B) in the catalytic core, compared with the original.

We subcloned the above PCR products (ribozymes) into the eucaryotic expression vector modified pHβApr-1 (pHβ-KrasRz, pHβ-disKrasRz), containing a selectable geneticin resistance gene (derived from pCEP4, Invitrogen, Figure 2) (Yamazaki et al, 1998). Expression of ribozymes or vectors was confirmed in the cells by reverse transcriptase-PCR (RT-PCR) with the following primers 5′-CGCTCGATGCGATGTTTCGC-3′ and 5′-CAATCG-GCTGCTCTGATGCC-3′. Specific PCR products were detected by Southern blotting with $^{32}$P-labelled oligonucleotide probes according to a simplified hybridization procedure.

### Introduction of anti-K-ras ribozymes in the colon cancer cells

Subconfluent SW480 cells were transfected with the pHβ-KrasRz or pHβ-disKrasRz by lipofecton (Lipofectin, Gibco BRL, Life Technologies, Gaithersburg, MD, USA). Stably transfected cells (SW480/KrasRz, SW480/disKrasRz) were selected for integration of vector in growth media containing 400 μg $ml^{-1}$ of G418 (geneticin) sulphate for 4 weeks (Sigma). G418-resistant colonies were grown and screened for expression of the ribozymes by RT-PCR assay with the following primers, 5′-AGCACAGAGC-CTCGCCTTT-3′ and 5′-GTCTGGATCCCTCGAAGC-3′, and probe 5′-CTCACGGACTCATCAGG-3′.

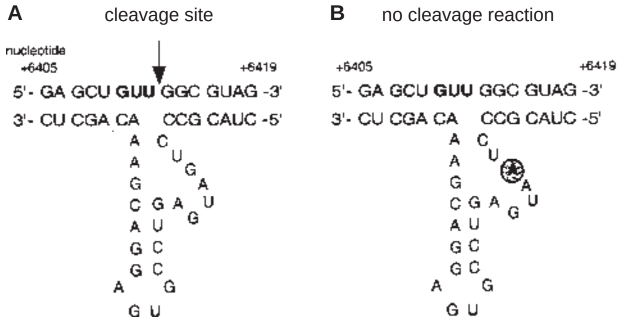

**Figure 1**(**A**) Schematic structure of substrate-ribozyme (KrasRz) complex. The arrowhead indicates the cleavage site. (**B**) The circled guanine nucleotide was changed to adenine in the disabled ribozyme (disKrasRz)

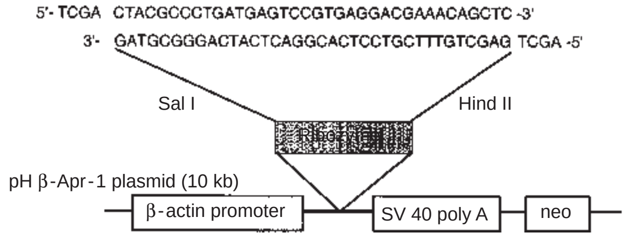

**Figure 2** Structures of expression plasmids pHβ-KrasRz and pHβ-disKrasRz introduced into SW480. The ribozyme and disabled ribozyme structures were cloned at the sites designated as 'PCR fragment'. The ribozyme sequences were driven by the β-actin promoter in the plasmids

### Cleavage of mutated K-ras oncogene in the colon cancer cells

Total cellular RNA fractions were extracted from the cancer cells and used for reverse transcription (1 μg total cellular RNA, 100 pM random primers, Boehringer Mannheim, Germany; reverse transcriptase, Gibco-BRL). T7 promoter-K-ras cDNA fragments were amplified by PCR with the following primers (Gene Amp PCR System 9600, Perkin Elmer: 5′-TAATACGACT-CACTATAGAACTTGTGGTAGTTGGAGCT-3′ and 5′-CTATTGTTGGATCATATTCG-3′). The ribozymes and substrate RNAs were prepared from the cDNA templates with T7 RNA polymerase (Stratagene, La Jolla, CA, USA) with α-$^{32}$P-UTP (111 TBq $mmol^{-1}$, Amersham) by in vitro transcription. Cleavage reactions were performed with these RNAs (substrate:ribozyme ratio = 1:100 at 37°C, $Mg^{2+}$ concentration = 0–40 mM; for 1 min–18 h in vitro. The cleavage products were detected by autoradiography (Kodak X-OMAT AR) (Scanlon et al, 1995).

### Angiogenesis-related factor gene expression

We examined the gene expression levels of various angiogenic factors in SW480/KrasRz and SW480/disKrasRz cells by Northern blotting analysis. Total RNA (20 μg) blots on nylon membranes (Gene Screen Plus, New England Nuclear) were

 © 2000 Cancer Research Campaign hybridized with $^{32}P$-labelled cDNA probes for VEGF-A, B and C, TSP-1 and 2 and BAI-1 as described previously (Tokunaga et al, 1998*a*; 1998*b*; 1999; Fukushima et al, 1998). The levels of VEGF-A and TSP1 gene expression were estimated by densitometry (Interactive Build Analysis System, Zeiss, Tokunaga et al, 1998*b*).

### Growth of colon cancer xenografts

The stable transformants (SW480/KrasRz cells, SW480/disKrasRz, SW480/vector; $5 \times 10^5$ cells) and parental cell line (SW480: $5 \times 10^5$ cells) were subcutaneously inoculated into nude mice ($n$ = 10 for each transformant and the parental cell line, female, 8 weeks, BALB/cA-*nu*, Clea Japan Inc., Tokyo). The growth rates of the cells were estimated by measuring the size of tumour lesions after inoculation. The size was calculated by the equation, $V = 0.5 \times A \times B^2$, in which A and B are the experimental measurements in mm of length and width, respectively.

All experiments on laboratory animals were performed in accordance with the care and use guidelines of the Central Institute for Experimental Animals.

### Histological examination of growth fraction in the colon cancer xenografts

We estimated growth fraction of the cells by bromodeoxyuridine (BrdU) incorporation. Tumours were removed from the mice under deep anaesthesia 2 h after intraperitoneal inoculation with 100 mg $kg^{-1}$ of BrdU (Sigma Chemical Company Ltd). Removed tumours were fixed in 10% formalin at room temperature for 24 h. Sections (3 µm thick) of the tumours were incubated with rat monoclonal anti-BrdU antibody (Sera-Lab. 1:40) for 45 min, after blocking of endogenous peroxidase, treatment with 2N-HCl for 30 min and 0.05% tyrosine for 15 min for retrieval of BrdU-antigenicity. The sections were then incubated with horseradish peroxidase (HRP)-conjugated rabbit anti-rat IgG (Dako, 1:200) for 30 min. Reaction products were visualized with 3,3′-diaminobenzidine with $H_2O_2$. The numbers of BrdU-positive cells were counted in three different visual fields under a light microscope at a magnification of × 450.

### Cytochemical analysis to observe apoptosis

A multistage process of apoptotic DNA fragmentation was confirmed by the terminal deoxynucleotidyl transferase (TdT)-mediated dUTP-biotin nick end-labelling method (TUNEL) using an ApopTaq Plus In Situ Apoptosis Detection Kit (Oncor, Gaithersburg, MD, USA). The SW480 cells on chamber slides were fixed with neutral-buffered formalin for 10 min, washed in phosphate-buffered saline (PBS), and quenched in 2% hydrogen peroxide for 5 min at room temperature. The specimens were then immersed in TdT buffer for 10 min at room temperature, reacted with a mixture of digoxigenin-labelled nucleotides and TdT enzyme for 60 min at 37°C, and treated with anti-digoxigenin antibody peroxidase conjugate for 30 min at room temperature. Apoptosis was detected by colour development using 3,3-diaminobenzidine tetrahydrochloride.

### Transmission electron microscopy

The frozen sections of tumours treated with modified Karnovsky solution (2% PFA, 0.5% glutaraldehyde, 40 mM phosphate buffer, pH 7.4, 150 mM NaCl) for 24 h, and post-fixed with 1% $OsO_4$ for 1 h at room temperature. The sections were dehydrated in ethanol and embedded in Quetol-812 (Nisshin EM, Tokyo, Japan). Ultrathin sections were stained with uranyl acetate and lead citrate, then examined with a transmission electron microscope (Model JEM-1200EX, JEOL, Tokyo, Japan).

### Statistical analysis

The statistical significance of differences in mean tumour volume and cell growth among the groups were examined by ANOVA, and significance was estimated by multiple comparison method according to Dunnett's test. The Fisher's exact test was applied for comparisons in xenotransplantability between group frequencies.

### RESULTS

### Cleavage of mutant K-ras oncogene transcripts in a cell-free system

To examine the enzymatic activity and specificity of this anti-K-ras ribozyme (KrasRz), we carried out in vitro cleavage reactions in a cell-free system (Figure 3). The KrasRz ribozyme specifically cleaved the GUU-mutated triplet at codon 12 of K-ras, which is present in the colon cancer cell line SW480. In vitro cleavage efficiency was dependent on the $Mg^{2+}$ concentration and the ribozyme/K-ras RNA ratio (data not shown). In contrast, KrasRz did not cleave the GGU wild-type triplet at codon 12 of K-ras derived from normal colonic mucosal cells. The disabled ribozyme (disKrasRz) did not cleave mutant K-ras RNA in vitro.

### Growth inhibition of the colon cancer cells by anti-K-ras ribozyme

In cell culture, these transformants showed similar morphological features to the parental cell line, while the ribozyme-transfectant (SW480/KrasRz) showed significantly decreased mitotic activity. The transformants were plated at a density of 5 × 10 000 cells per dish. For generation time assay, experiments were performed twice in duplicate. Growth of SW480/KrasRz was significantly suppressed in culture. In contrast, the SW480/disKrasRz and SW480 cells did not show any significant growth suppression. Generation time of the SW480/KrasRz cells (49 h) was significantly prolonged by 2.1-fold compared to the SW480 cells (24 h) (Figure 4A, $P < 0.01$, Dunnett's test).

To determine the effects of KrasRz on tumour formation, SW480/KrasRz cells were inoculated into nude mice ($5 \times 10^5$ cells per mouse). Only two tumours (2/10) had formed 25 days after inoculation with XeSW480/KrasRz, whereas tumours formed in almost all mice inoculated with XeSW480/disKrasRz (10/10) and the SW480 parental cell transfected with vector alone (10/10) and the SW480 parental cell line (8/10) ($P < 0.05$, Fisher's exact test). The tumour volume of XeSW480/KrasRz (305 $mm^3$) was significantly decreased as compared with XeSW480/disKrasRz (980 $mm^3$) and the parental cells (1095 $mm^3$) (on day 42 after inoculation, $5 \times 10^5$ cells per mouse, $P < 0.01$, Dunnett' test, Figure 4B). The BrdU incorporation was significantly decreased in the XeSW480/KrasRz transformants (11 ± 12) compared to XeSW480/disKrasRz (32 ± 15) and the parental cells (41 ± 19) ($P < 0.05$, Dunnett' test, Figure 5A,B).

© 2000 Cancer Research Campaign

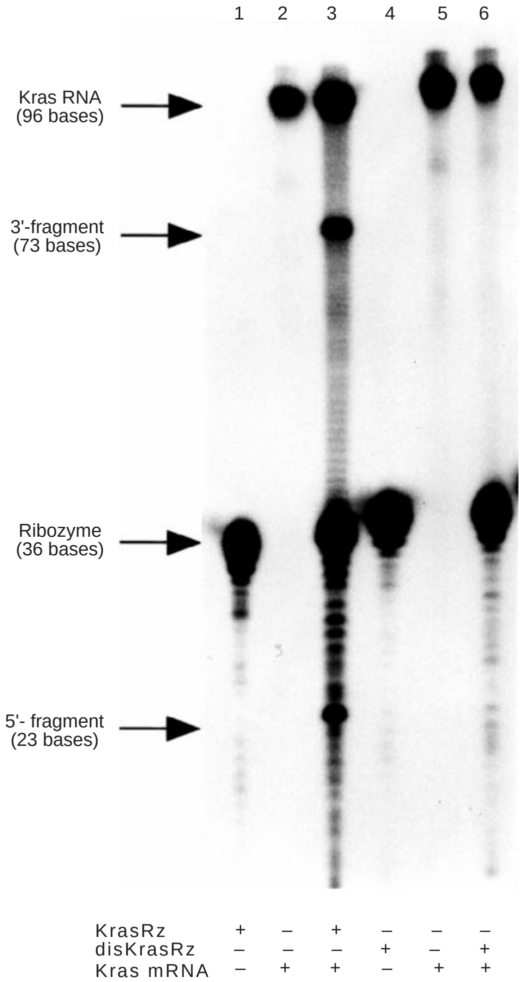

**Figure 3** Cleavage reaction of the K-ras RNA by ribozyme in vitro. Cleavage activity of the ribozyme targeting the mutated K-ras RNA. The ribozyme and fragment RNA were labelled with α-$^{32}$P-UTP (111 TBq mmol$^{-1}$). Lane 1, anti-Kras Rz only (36 bases); Lane 2, K-ras RNA originated by SW480 (96 bases) only; Lane 3, both anti-Kras Rz and Kras RNA. The anti-Kras Rz cleaved K-ras RNA into two products, a 23-base 5′-fragment and a 73-base 3′-fragment. Lane 4, anti-disKras Rz only (36 bases); Lane 5, K-ras RNA from SW480 (96 bases) only; Lane 6, both anti-disKras Rz and K-ras RNA. The anti-disKras Rz did not cleave K-ras RNA

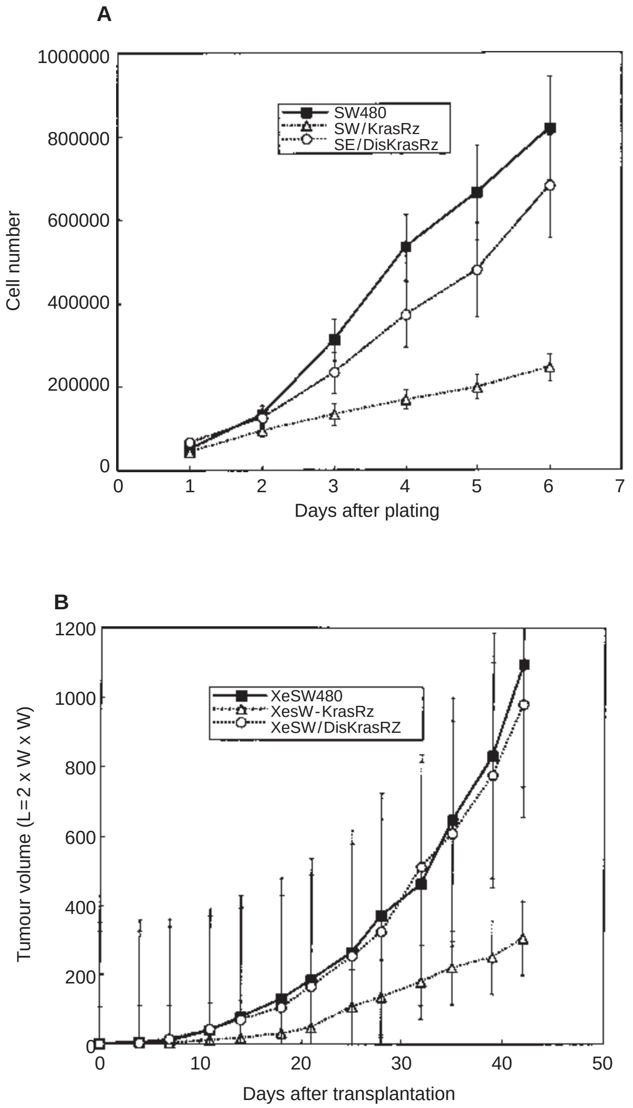

**Figure 4** Growth inhibition mediated by anti-KrasRz. (**A**) Relative growth of colon cancer cell lines in culture. The cell lines were plated at $5 \times 10^5$. The cell growth data shown are means ± SD of five independent plates. Each sample was counted in triplicate. Differences among the cell lines were determined by ANOVA. The growth rate of SW480/KrasRz was significantly lower than those of SW480/disKrasRz or SW480 cells ($P < 0.01$, Dunnett's test). (**B**) Tumour volume of the xenografts of the cell lines. The tumour volume (mm$^3$) was determined as follows; $0.5 \times$ length $\times$ width$^2$. The volume of XeSW480/KrasRz tumours was significantly less than those of XeSW480/disKrasRz or XeSW480 ($P < 0.01$, Dunnett's test)

### Alteration of angiogenic factor gene expression by anti-K-ras ribozyme

We evaluated the expression of various angiogenic factors by Northern blotting analysis with total cellular RNA preparation (20 μg). Relative expression level was calculated by the ratio $A_1$:$A_0$, where each $A_0$ and $A_1$ is the gene expression of parental cell or transformant respectively, standardized by rehybridization of the β2-microglobulin gene in each cell line. The SW480/KrasRz cells showed almost 90% reduction in levels of VEGF-A gene expression compared to SW480/parental cells, while the expression was unchanged in SW480/disKrasRz (Figure 6A). The level of VEGF-A-gene expression was unchanged in SW480 cells transfected with pHβApr-1 vector (data not shown). The cell lines showed no marked changes in levels of VEGF-B or C gene expression. SW480/KrasRz cells also showed an almost 80% increase in expression of the angiostatic factor TSP1 as compared with SW480/disKrasRz and parental SW480 cells (Figure 6B). No expression of the other angiostatic factors TSP2 and BAI-1 was detected in these transformants.

### Apoptosis induced by inactivation of mutant K-ras oncogene

Oligonucleosomal DNA fragmentation or condensation was observed in cells treated with anti-KrasRz (Figure 7). The cells

*© 2000 Cancer Research Campaign*

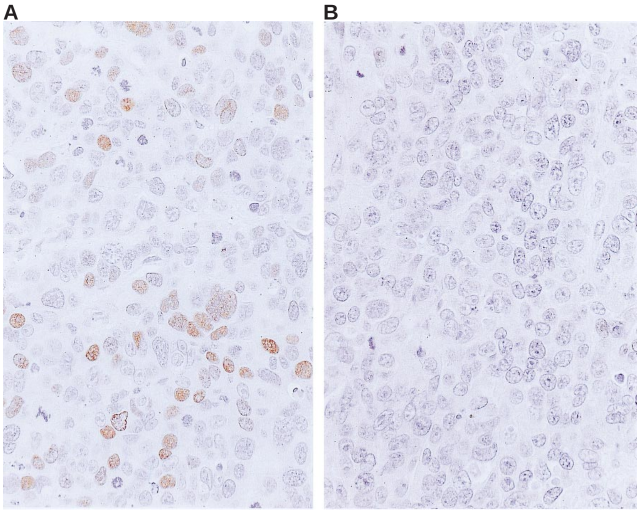

**Figure 5** BrdU immunoreactivity in the xenografted tumour cells. BrdU incorporation in Xe480/disKrasRz (**A**) was more than that in Xe480/KrasRz (**B**) (× 450)

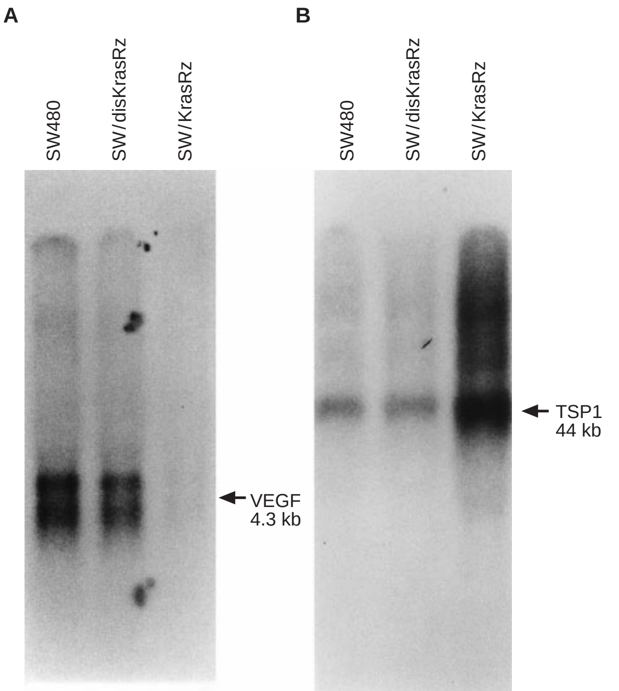

**Figure 6** Expression of angiogenic-related gene in the cell lines. (**A**) VEGF gene (**B**) TSP1 gene. Lane 1, SW480/KrasRz cells; Lane 2, SW480/disKrasRz cells; Lane 3, SW480 cells. The SW480/KrasRz cells showed suppressed level of VEGF gene expression compared with SW480/disKrasRz or SW480 cells, while the SW480/KrasRz cells showed increased level of TSP1 gene expression compared with SW480/disKrasRz or SW480 cells with DNA fragmentation were DAB-positive due to TdT reactions. In contrast, few apoptotic cells were noted when the cells were treated with dis-KrasRz.

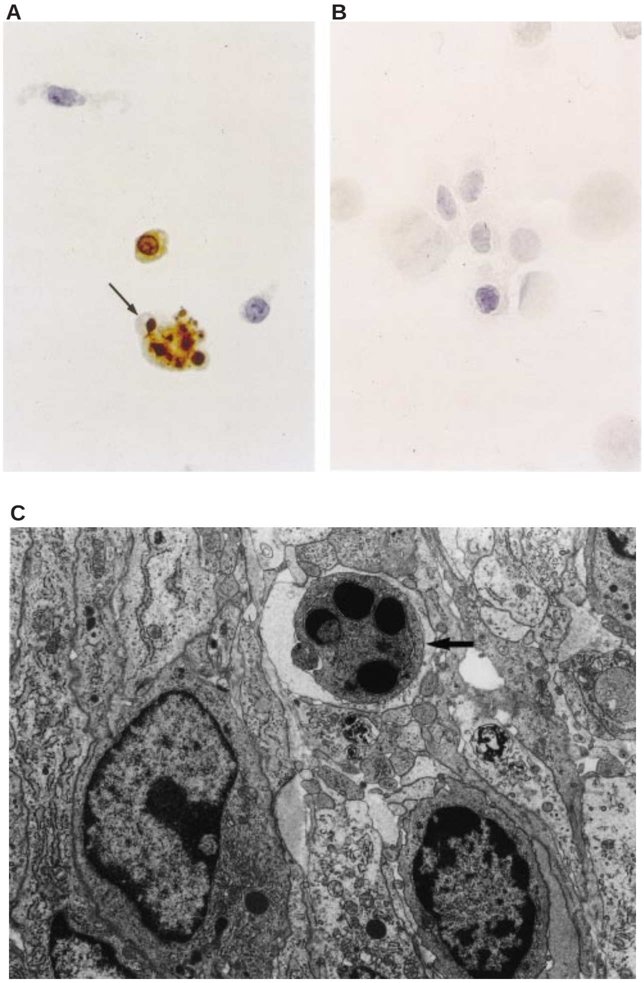

**Figure 7** Histological features of apoptotic cells mediated by anti-KrasRz. (**A**) The SW480/KrasRz cells showed DAB-positive due to TdT reactions (**B**) while the SW480/disKrasRz cells did not (× 150). (**C**) Xe480/KrasRz showed nucleosomal DNA fragmentation (arrow) and apoptotic body by electron microscopy (× 4000)

### DISCUSSION

In this study, we constructed a hammerhead-type ribozyme (KrasRz) to cleave mutated K-ras mRNA transcripts in vitro and in vivo. KrasRz specifically cleaved mutant K-ras RNA in a cell-free system. We evaluated the biological effects of KrasRz in the human colonic cancer cell line SW480. Our results demonstrated that KrasRz effectively cleaved the mutant K-ras mRNA and significantly suppressed growth of this colonic cancer cell line. Inhibition of the function of mutant ras alleles in cancer cells by treatment with farnesyltransferase inhibitors suppresses the growth of solid tumours in vivo (Kohl et al, 1993). Disruption of mutant K-ras activation by triplet DNA was associated with growth alteration of colon cancer cell lines (Shirasawa et al, 1993). We showed here that the inactivation of mutant K-ras mRNA by a specific hammerhead-type ribozyme could suppress growth of a human colonic cancer cell line in vitro and in vivo.

Angiogenesis is a major mechanism involved in the growth and metastasis of solid tumour lesions. We reported previously that various kinds of angiogenesis-related factors play important roles in malignant features including tumour size, stromal vascularity and distant metastasis of various cancers (Tokunaga et al, 1998*a*; Tomisawa et al, 1999; Tsuchida et al, 1999). Expression of the cell-associated isoform of VEGF-A, VEGF189, was significantly correlated with liver metastasis in colonic cancers (Tokunaga et al, 1998*a*). VEGF189 expression was also correlated with growth of non-small cell lung cancer and renal cell carcinoma (Oshika et al, 1998; Tomisawa et al, 1999). On the other hand various angiostatic factors affect the vascularization and growth of solid tumours (Oshika et al, 1999; Tokunaga et al, 1999). TSP1 expression was correlated with tumour size of colonic cancer (Tokunaga et al, 1998*b*). These studies suggested the VEGF-A and TSP were major factors involved in vascularization and growth of human solid tumours.

In this study, attenuation of activated K-ras oncogene by a hammerhead-type ribozyme, KrasRz, decreased VEGF-A gene expression in the colonic cancer cell line SW480 (SW480/KrasRz), but did not affect VEGF-B or C gene expression. VEGF-A gene expression of the angiostatic factor TSP1 was simultaneously increased in SW480/KrasRz. These alterations of angiogenic phenotype induced by the ribozyme were responsible for reduced vascularization of the tumour. It has been reported that angiogenic phenotype was affected by the accumulation of sequential mutations in multiple genes, including the activation of oncogenes or the ablation of a tumour suppressor gene (Rak et al, 1995; Volpert et al, 1997). Production of VEGF is stimulated by ras in NIH3T3 fibroblasts and in intestinal cell lines (Grugel et al, 1995; Rak et al, 1995). Transfection with activated ras decreased the level of inhibitory TSP1 in bronchial epithelial cells (Zabrenetzky et al, 1994). Wild-type p53 downregulated endogenous VEGF mRNA in a human glioma cell line and a human fetal kidney cell line (Mukhopadhyay et al, 1995). We also reported that accumulation of p53 protein was inversely correlated with TSP1 expression (Tokunaga et al, 1998*b*). These results suggested that alteration of angiogenesis-related factor expression was achieved by modulation of expression of various oncogenes or oncosuppressor genes. The downregulation of mutant K-ras oncogene by anti-Kras ribozyme (KrasRz) significantly suppressed angiogenic features with decreased VEGF-A and increased TSP1, whereas expression of other angiogenic factors (VEGF-B and C) and angiostatic factors (TSP2 and BAI-1) was not affected. The mechanisms responsible for the regulation of angiogenic features are complex.

The ras gene seems to be a good candidate for targeting of oncogene modulation by ribozymes. Anti-ras ribozymes have been tested in several tumour types. Anti-H-ras ribozyme showed marked growth inhibition and suppression of tumorigenicity in a bladder carcinoma cell line (Feng et al, 1995). The bladder carcinoma cell line transfected with anti-H-ras ribozyme showed limited invasion and longer overall survival. The attenuation of K-ras gene expression by anti K-ras ribozyme inhibited growth of a pancreatic cell line. In this study, anti-K-ras ribozyme (KrasRz) showed similar effects on a colonic cancer cell line with a K-ras mutation. The results presented here indicate that mutant K-ras gene increases the growth of colon cancer cells by reduction of apoptosis and enhancement of angiogenesis in vivo. We demonstrated that an anti-K-ras ribozyme induced apoptosis in a pancreatic cancer cell line (Tsuchida et al, 2000). In this study, we demonstrated apoptotic changes induced by the anti-K-ras ribozyme in a colon cancer cell line. The results suggested that the ribozyme-mediated inactivation of mutant K-ras oncogene resulted in growth suppression, via apoptosis of various cancer cells, and a shift in the angiostatic conditions in the cancer stroma.

Reduced proliferation seemed to be a major factor affecting growth reduction of the SW480/KrasRz. The mitotic activity or fractions of BrdU-positive cells were clearly decreased in SW480/KrasRz in the xenografts compared to SW480/disKrasRz, while the apoptotic changes were definitive in certain cells in SW480/KrasRz. The tumour growth suppression by the anti-K-ras ribozyme was achieved by a combination of the above factors. Further analysis and improvements are required for therapeutic application of the anti-K-ras ribozyme (KrasRz) in human colon cancer. Such studies may lead to new therapeutic strategies especially for management of vascular invasion and distant metastasis of colon cancer.

### ACKNOWLEDGEMENTS

This work was supported in part by Grants-in-Aid for Scientific Research from the Ministry of Education, Science and Culture of Japan and Tokai University School of Medicine Research Project. We thank Mr Yuichi Tada and Ms Kyoko Murata for their technical assistance.

### REFERENCES

Andreyev HJ, Norman AR, Cunningham D, Oates JR and Clarke PA (1998) Kirsten ras mutations in patients with colorectal cancer: the multicenter 'RASCAL' study. *J Natl Cancer Inst* **90**: 675–684

Bouffard DY, Ohkawa T, Kijima H, Irie A, Suzuki T, Curcio LD, Holm PS, Sassani A and Scanlon KJ (1996) Oligonucleotide modulation of multidrug resistance. *Eur J Cancer* **32A**: 1010–1018

Cech TR, Zaug AJ and Grabowski PJ (1981) *In vitro* splicing of the ribosomal RNA precursor of tetrahymena: Involvement of a guanosine nucleotide in the excision of the intervening sequence. *Cell* **27**: 487–496

Chi S, Kitanaka C, Noguchi K, Mochizuki T, Nagashima Y, Shirouzu M, Fujita H, Yoshida M, Chen W, Asai A, Himeno M, Yokoyama S and Kuchino Y (1999) Oncogenic Ras triggers cell suicide through the activation of a caspase-independent cell death program in human cancer cells. *Oncogene* **18**: 2281–2290

Cho KR and Vogelstein B (1992) Genetic alterations in the adenoma carcinoma sequence. *Cancer* **70**: 1727–1731

Feng M, Cabrera G, Deshane J, Scanlon KJ and Curiel DT (1995) Neoplastic reversion accomplished by high efficiency adenoviral-mediated delivery of an anti-ras ribozyme. *Cancer Res* **55**: 2024–2028

Fukushima Y, Oshika Y, Tsuchida T, Tokunaga T, Hatanaka H, Kijima H, Yamazaki H, Ueyama Y, Tamaoki N and Nakamura M (1998) Brain-specific angiogenesis inhibitor 1 expression is inversely correlated with vascularity and distant metastasis of colorectal cancer. *Int J Oncol* **13**: 967–970

Grugel S, Finkenzeller G, Weindel K, Barleon B and Marme D (1995) Both v-Ha-Ras and v-Raf stimulate expression of the vascular endothelial growth factor in NIH 3T3 cells. *J Biol Chem* **270**: 25915–25919

Hsu SC, Volpert OV, Steck PA, Mikkelsen T, Poverini PJ, Rao S, Chou P and Bouck N (1996) Inhibition of angiogenesis in human glioblastomas by chromosome 10 induction of thrombospondin-1. *Cancer Res* **56**: 5684–5691

Kieser A, Weich HA, Brandner G, Marme D and Kolch W (1994) Mutant p53 potentiates protein kinase C induction of vascular endothelial growth factor expression. *Oncogene* **9**: 964–969

Kijima H, Ishida H, Ohkawa T, Kashani-Sabet M and Scanlon KJ (1995) Therapeutic applications of ribozymes. *Pharmacol Ther* **68**: 247–267

Kijima H, Bouffard DY and Scanlon KJ (1998) Ribozymes as a novel approach for the treatment of human pancreatic carcinoma. *Methods Mol Med* **11**: 193–208

Jednak MA and Nostrant TT (1998) Screening for colorectal cancer. *Prim Care* **25**: 293–308

Kohl NE, Mosser SD, Desolmes SJ, Giuliani EA, Pompliano DL, Graham SL, Smith RL, Scolnick EM, Oliff A and Gibbs JB (1993) Selective inhibition of ras-dependent transformation by a farnesyltransferase inhibitor. *Science* **260**: 1934–1942

© 2000 Cancer Research Campaign

Matsuguchi T and Kraft AS (1998) Regulation of myeloid cell growth by distinct effectors of Ras. *Oncogene* **17**: 2701–2709

Mukhopadhyay D, Tsiokas L and Sukhatme PV (1995) Wild-type p53 and v-Src exert opposing influences on human vascular endothelial growth factor gene expression. *Cancer Res* **55**: 6161–6165

Ohta Y, Kijima H, Kashani-Sabet M and Scanlon KJ (1996) Tissue-specific expression of an anti-ras ribozyme inhibits proliferation of human malignant melanoma cells. *Nucleic Acids Res* **24**: 938–942

Okada F, Rak J, Croix B, Lieubeau B, Kaya M, Rncari L, Shirasawa S, Sasazuki T and Kerbel R (1998) Impact of oncogenes in tumor angiogenesis: Mutant K-ras up-regulation of vascular endothelial growth factor/vascular permeability factor is necessary, but not sufficient for tumorigenicity of human colorectal carcinoma cells. *Proc Natl Acad Sci USA* **95**: 3609–3614

Oshika Y, Masuda K, Tokunaga T, Hatanaka H, Kamiya T, Abe Y, Ozeki Y, Kijima H, Yamazaki H, Tamaoki N, Ueyama Y, Nakamura M (1998) Thrombospondin2 gene expression is correlated with decreased vascularity in non-small cell lung cancer. *Clin Cancer Res* **4**: 1785–1788

Oshika Y, Nakamura M, Tokunaga T, Ozeki Y, Fukushima Y, Hatanaka H, Abe Y, Yamazaki H, Kijima H, Tamaoki N and Ueyama Y (1998) Expression of cell-associated isoform of vascular endothelial growth factor 189 and its prognostic relevance in non-small cell lung cancer. *Int J Oncol* **12**: 541–544

Rak J, Mitsuhashi Y, Bayko L, Filmus J, Shirasawa S, Sasazuki T and Kerbel RS (1995) Mutant ras oncogenes upregulate VEGF/VPF expression: implications for induction and inhibition of tumor angiogenesis. *Cancer Res* **55**: 4575–4580

Saraga E, Bautista D, Dorta G, Chaubert P, Martin P, Sordat B, Protiva P, Blum A, Bosman F and Benhattar J (1997) Genetic heterogeneity in sporadic colorectal adenomas. *J Pathol* **181**: 281–286

Scanlon KJ, Jiao L, Funato T, Wang W, Tone T, Rossi JJ and Kashani-Sabet M (1991) Ribozyme-mediated cleavage of c-fos mRNA reduces gene expression of DNA synthesis enzymes and metallothionein. *Proc Natl Acad Sci USA* **88**: 10591–10595

Scanlon KJ, Ohta Y, Ishida H, Kijima H, Ohkawa T, Kaminski A, Tsai J, Horng G and Kashani-Sabet M (1995) Oligonucleotide-mediated modulation of mammalian gene expression. *FASEB J* **6**: 1288–1296

Siemeister G, Weindel K, Mohrs K, Barleon B, Martiny G and Marme D (1996) Reversion of deregulated expression of vascular endothelial growth factor in human renal carcinoma cells by von Hippel-Lindau tumor suppressor protein. *Cancer Res* **56**: 2299–2301

Shirasawa S, Furuse M, Yokoyama N and Sasazuki T (1993) Altered growth of human colon cancer cell lines disrupted at activated Ki-ras. *Science* **260**: 85–88

Tokunaga T, Oshika Y, Abe Y, Ozeki Y, Sadahiro S, Kijima H, Tsuchida T, Yamazaki H, Ueyama Y, Tamaoki N and Nakamura M (1998*a*) Vascular endothelial growth factor (VEGF) mRNA isoform expression pattern is correlated with liver metastasis and poor prognosis in colon cancer. *Br J Cancer* **77**: 998–1002

Tokunaga T, Nakamura M, Oshika Y, Tsuchida T, Kazuno M, Fukushima Y, Kawai K, Abe Y, Kijima H, Yamazaki H, Tamaoki N and Ueyama Y (1998*b*) Alterations in tumour suppressor gene p53 correlate with inhibition of thrombospondin-1 gene expression in colon cancer cells. *Virchows Arch* **433**: 415–418

Tokunaga T, Nakamura M, Oshika Y, Abe Y, Ozeki Y, Fukushima Y, Sadahiro S, Kijima H, Tsuchida T, Yamazaki H, Tamaoki N and Ueyama Y (1999) Thrombospondin-2 expression is correlated with inhibition of angiogenesis and metastasis of colon cancer. *Br J Cancer* **79**: 354–359

Tomisawa M, Tokunaga T, Oshika Y, Tsuchida T, Fukushima Y, Sato H, Kijima H, Yamazaki H, Ueyama Y, Tamaoki N, Nakamura M (1999) Expression of vascular endothelial growth factor isoform is closely correlated with tumor stage and vascularisation in renal cell carcinoma. *Br J Cancer* **35**: 133–137

Tsuchida T, Kijima H, Oshika Y, Tokunaga T, Abe Y, Yamazaki H, Tamaoki N, Ueyama Y, Scanlon KJ and Nakamura M (1998) Hammerhead ribozyme specifically inhibits mutant K-ras mRNA of human pancreatic cancer cells. *Biochem Biophys Res Common* **253**: 368–378

Tsuchida T, Kijima H, Tokunaga T, Oshika Y, Hatanaka H, Fukushima Y, Abe Y, Kawai K, Yoshida Y, Miura S, Yamazaki H, Tamaoki N, Ueyama Y and Nakamura M (1999) Expression of the thrombospondin 1 receptor CD36 is correlated with decreased stromal vascularisation in colon cancer. *Int J Oncol* **14**: 47–51

Tsuchida T, Kijima H, Hori S, Oshika Y, Tokunaga T, Kawai K, Yamazaki H, Ueyama Y, Scanlon KJ, Tamaoki N and Nakamura M (2000) Adenovirus-mediated anti-K-ras ribozyme induces apoptosis and growth suppression of human pancreatic carcinoma. *Cancer Gene Ther* (in press)

Volpert OV, Dameron KM and Bouck N (1997) Sequential development of an angiogenic phenotype by human fibroblasts progressing to tumorigenity. *Oncogene* **14**: 1495–1502

Ward RL, Todd AV, Santiago F, O'Connor T and Hawkins NJ (1997) Activation of the K-ras oncogene in colorectal neoplasms is associated with decreased apoptosis. *Cancer* **79**: 1106–1113

Yamazaki H, Kijima H, Ohnishi Y, Abe Y, Oshika Y, Tsuchida T, Tokunaga T, Tsugu A, Ueyama Y, Tamaoki N, Nakamura M (1998) Inhibition of tumor growth by ribozyme-mediated suppression of aberrant epidermal growth factor receptor gene expression. *J Natl Cancer Inst* **90**: 581–587

Zabrenetsky V, Harris CC, Steeg PS and Roberts DD (1994) Expression of the extracellular matrix molecule thrombospondin inversely correlates with malignant progression in melanoma, lung, and breast carcinoma cell lines. *Int J Cancer* **59**: 191–195

© 2000 Cancer Research Campaign
